# A novel protocol to isolate, detect and differentiate taeniid eggs in leafy greens and berries using real-time PCR with melting curve analysis

**DOI:** 10.1186/s13071-019-3834-8

**Published:** 2019-12-18

**Authors:** Caroline F. Frey, Jenna R. Oakley, Vladislav A. Lobanov, Nelson Marreros, Janna M. Schurer, Laura F. Lalonde

**Affiliations:** 1 Centre for Food-borne and Animal Parasitology, Canadian Food Inspection Agency, Saskatoon, SK S7N 2R3 Canada; 2 Parks Canada Agency, 52 Campus Drive, Saskatoon, SK S7N 5B4 Canada; 3 Western College of Veterinary Medicine, Department of Veterinary Microbiology, University of Saskatchewan, Saskatoon, SK Canada; 4 Cummings School of Veterinary Medicine at Tufts University, North Grafton, MA USA; 5 University of Global Health Equity, Kigali, Rwanda

**Keywords:** *Echinococcus* spp., *Taenia* spp., Leafy greens, Berries, Food safety

## Abstract

**Background:**

Zoonotic taeniid cestodes are amongst the most important food-borne parasites affecting human health worldwide. Contamination of fresh produce with the eggs of *Echinococcus granulosus* (*s.l.*), *Echinococcus multilocularis*, and some *Taenia* species pose a potential food safety risk. However, very few studies have attempted to investigate the potential contamination of fresh produce with taeniid eggs and the available methods are not standardized for this purpose. Established protocols do exist for testing leafy greens and berries for contamination with protozoan parasites and are used in national surveillance programmes. This methodology could be suitable for the detection of taeniids. The objective of this project was to develop and standardize a sensitive and reliable method to detect contamination of leafy greens and berries with eggs of zoonotic taeniids and to differentiate between *E. multilocularis*, *E. granulosus* (*s.l*.) and *Taenia* spp.

**Methods:**

We compared the efficacy of different wash solutions to remove *Taenia* spp. eggs from spiked produce, assessed two DNA extraction kits for their performance on *Taenia* spp. eggs, and adapted a published conventional multiplex PCR into a real-time PCR with fluorescence melting curve analysis (MCA) that was optimized for use on produce washes. Analytical specificity of this protocol was assessed using non-spiked produce washes as well as a variety of other potentially contaminating parasites.

**Results:**

The protocol as established in this study had an analytical sensitivity of detecting five eggs per spiked sample for both romaine lettuce and strawberries. Unequivocal identification of *E. multilocularis*, *E. granulosus* (*s.l*.) and *Taenia* spp. was possible through MCA. Amplicon sequencing allowed identification of *Taenia* to the species level. The real-time PCR also amplified DNA from *Dicrocoelium* sp., but with a clearly discernable melting curve profile.

**Conclusion:**

The new protocol for screening produce for taeniid contamination was highly sensitive. Melting curve analysis and the possibility of amplicon sequencing made this assay very specific. Once further validated, this method could be employed for surveillance of produce for contamination with taeniid parasites to assess potential risks for consumers.
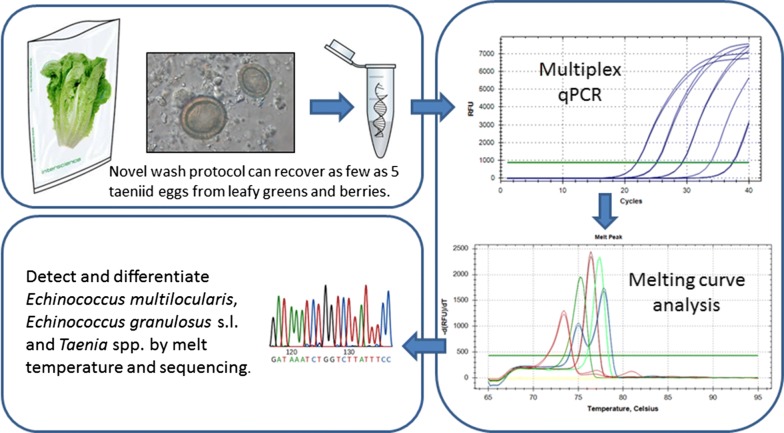

## Background

Parasites in food are an increasing concern for scientific and regulatory bodies [1]. More specifically, fresh produce contaminated with eggs of *Echinococcus multilocularis* or *E. granulosus* has been identified among the top priorities in the field of food-borne parasites [2, 3]. Also, some *Taenia* species (e.g. *Taenia solium*), can cause serious infection in humans if the eggs are ingested. Humans consuming presumably even low numbers of these eggs contaminating fresh vegetables, fruits, and berries could become infected and develop cystic or alveolar echinococcosis or metacestode infection with *Taenia* spp. (e.g. cysticercosis). Such infections can be disabling and potentially fatal if left untreated [4]. While food-borne transmission of taeniids is well recognized [1–3, 5], there have been only a few studies to elucidate the risk posed by contamination of produce with taeniid eggs [6, 7], or more specifically with *E. multilocularis* eggs [8, 9]. The rarity of such studies may be explained by the lack of standardized and validated methods to test produce for taeniid contamination. Nevertheless, further studies to address these potential risks would be highly desirable [5].

Several challenges need to be mitigated when testing produce for parasite contamination. First, the usually sticky parasite eggs or oocysts have to be reliably removed from the produce matrix. Different wash solutions have been described for this purpose, such as tap water [6], 0.85% sodium chloride solution [7], Tween-80 [8], the laboratory detergent 0.1% Alconox [10], sodium pyrophosphate solution [11] or glycine [12]. Secondly, the parasites must be efficiently concentrated and isolated from the produce wash because no methods currently exist for laboratory enrichment of parasites from food samples, such as those available for bacteria. Methods such as filtration/sieving [6, 8], sedimentation and/or centrifugation [7, 8, 13], as well as flotation [8, 14], have previously been used for this purpose. Identification of the parasite eggs can subsequently be accomplished by traditional light microscopy [7] or by molecular methods [6, 8, 13, 14]. In the case of taeniids, the eggs of *Echinococcus* spp. and *Taenia* spp. are not morphologically discernable; thus, identification relies on molecular methods [6]. The DNA extraction method must be able to break open the thick-walled eggs and to remove inhibitors present in environmental samples [13, 15]. Finally, the molecular method of choice should detect all taeniids of interest and discriminate between *E. multilocularis*, *E. granulosus* (*s.l*.) and *Taenia* spp. The method should be sufficiently specific to perform reliably on difficult matrices such as produce wash, where soil and environmental microbes such as fungi and bacteria are present [13]. Last but not least, the procedure must have a high sensitivity to detect the low levels of contamination that are expected to occur on produce intended for human consumption [16].

Well-established and validated methods are available to test produce for the presence of zoonotic protozoan parasites [12–14, 17]. These methods are currently used in regulatory surveillance activities in Canada and the USA [13, 14]. The goal of the present study was to develop and standardize a protocol for the reliable and sensitive detection and identification of taeniid contamination of fresh produce based on established protocols for protozoal contamination of similar matrices.

## Methods

### Produce samples

Romaine lettuce and strawberries were purchased from local retail supermarkets in Saskatoon, SK, Canada. Each batch of produce was screened for pre-existing taeniid contamination by applying the washing, extraction and PCR protocol as described below.

### Parasites

Adult *Taenia pisiformis* (*n* = 3), *T. hydatigena* (*n* = 1) and *E. granulosus* (G8/G10) (*n* = 3 different wolves, *Canis lupus*) were obtained from intestines of wolves sampled in eastern North America [18]. Adult *E. multilocularis* worms (*n* = 1, coyote *Canis latrans*) were obtained from the intestines of a coyote from SK, Canada (provided by Dr E. J. Jenkins, University of Saskatchewan). All intestines containing the adult worms were frozen at − 80 °C for 5 days and the recovered worms were subsequently stored in 70% ethanol. Fresh DNA from *T. saginata* was isolated from archived *Cysticercus bovis* [19].

Analytical specificity of the assay was assessed using produce spiked with large numbers (100 to 500 cysts or oocysts per sample) of *Eimeria papillata* (*n* = 4), *Giardia duodenalis* (*n* = 4), and *Cyclospora cayetanensis* (*n* =2) as well as non-spiked produce (*n* = 50). In addition, genomic DNA (gDNA) of *Toxoplasma gondii* types I (*n* = 1), II (*n* = 1), III (*n* = 1) and atypical (*n* = 1), *Sarcocystis* sp. (*n* = 5), *Uncinaria* sp. (*n* = 2), *Toxascaris* sp. (*n* = 17), *Capillaria* sp. (*n* = 3), *Trichuris* sp. (*n* = 1), *Alaria* sp. (*n* = 30), and *Dicrocoelium* sp. (*n* = 1) was used to assess the analytical specificity of the real-time PCR-MCA.

### Spiking of produce samples

Spiking experiments were performed using *T. pisiformis* eggs as a surrogate for all taeniid eggs. Gravid proglottids of the adult worm were sectioned with sterile blades and mixed with phosphate-buffered saline (PBS). Eggs were subsequently purified by passing the sectioned proglottids through a 100 µm filter and then stored in sterile PBS with added 1× antibiotic-antimycotic solution (Sigma-Aldrich, St. Louis, MO, USA). Eggs were counted on a gridded deep-well slide and selected based on their morphology before each spiking experiment. If eggs appeared cracked or their morphology was atypical, fresh eggs were prepared from another proglottid. Aliquots (25 µl) of each of the diluted spiking stocks were counted in duplicate to confirm the accuracy of the number of eggs pipetted onto the produce. Romaine lettuce samples (35 g each) were spiked with 25 µl aliquots containing 500 (*n* = 30 samples), 100 (*n* = 16), 50 (*n* = 15), 10 (*n* = 12) or 5 eggs (*n* = 21). Strawberry samples (55 g each) were similarly spiked with 100 (*n* = 18), 50 (*n* = 1), 10 (*n* = 9) or 5 eggs (*n* = 10). Eggs were spiked in 3–4 droplets directly onto produce samples already placed in the filter bags, using a 100 µl air displacement pipette fitted with a plastic filter tip, and the prepared samples were subsequently incubated overnight at 4 °C prior to processing.

### Wash protocol

To wash the samples, 100 ml of 0.1% Alconox [10, 13], 1 M Glycine pH 5.5 [12], or sodium pyrophosphate solution (0.563 mM H_2_Na_2_P_2_O_7_, 42.8 mM NaCl) [11] was added to the filter bag. The air was carefully removed and the bag was secured with a clip. Each bag was then laid flat on an orbital shaker and shaken for 30 min at 85× *rpm*. Bags were flipped over at 15 min to ensure complete submersion of the produce. Subsequently, fluid from the filtrate side of the bags was aspirated and transferred into a 250 ml conical centrifuge tube using a 25 ml polystyrene serological pipet. Tubes were centrifuged at 2000×*g* for 15 min at room temperature using a low brake setting. Supernatants were discarded by vacuum aspiration to a residual volume of about 5 ml. Simultaneously, the samples in the filter bags were rinsed with an additional 100 ml of the respective wash solution. After rinsing, liquid was aspirated from each bag and added to the 250 ml tubes using a serological pipette. Centrifugation was repeated as before. Afterwards, the pellet was re-suspended and transferred to a 15 ml tube using a glass pipet. The 250 ml tubes were rinsed with 2 ml deionised water and this was added to the pellet in the 15 ml tube. The 15 ml tube was then centrifuged at 2000×*g* for 20 min using a low brake setting. The supernatant was discarded to twice the pellet volume, which was transferred using a glass pipette to a 2 ml tube and then centrifuged for 4 min at 14,000×*g*. The supernatant was removed without disturbing the pellet, resulting in a final volume of twice the pellet size, i.e. between 100–800 µl. The pellet was stored at 4 °C for up to five days prior to DNA extraction.

### DNA extraction

DNA was extracted from concentrated produce washes using either the FastDNA™ SPIN Kit for Soil and the FastPrep™-24 Instrument (MP Biomedicals, Santa Ana, CA, USA) or the QIAamp® DNA Stool Mini Kit (Qiagen, Hilden, Germany). The QIAamp® DNA Stool Mini Kit was used with significant modifications made to the manufacturer’s instructions, as reported previously for the detection of *Cyclospora* DNA in human stool samples [20]. We incorporated 8 freeze-thaw cycles (liquid nitrogen and 95 °C water bath) after the addition of ASL buffer followed by the addition of 20 µl of proteinase K, and incubation at 56 °C for 3 h. The provided InhibitEX tablet was added, followed by vortexing the solution for 1 min. The InhibitEX matrix was removed by centrifugation, and 200 µl of AL buffer was added to the supernatant. From this step onwards, the manufacturer’s instructions were followed. The FastDNA™ SPIN Kit for Soil was also modified, in brief, lysing matrix E provided in the kit was added to the pellet of the produce wash and subsequent steps were exactly as described by Murphy et al. [13], with two exceptions: 5 ml tubes were used for the binding reaction instead of 15 ml tubes and inverting was performed using a rotator at a speed of 30 inversions per min instead of manually. DNA was eluted by adding 2 × 50 µl DNase/pyrogen-free water (DES from the supplied kit) to the silica matrix followed by centrifugation at 14,000×*g* for 1 min. For both extraction methods, DNA samples were stored at 4 °C for up to four days, or at − 20 °C for longer periods. Negative (water and kit reagents) and positive (*T. pisiformis* eggs in negative produce wash) extraction controls were included in all extractions.

### Real-time PCR-MCA

Primers used were originally published by Trachsel et al. [21]. Primers Cest 1 (5′- TGC TGA TTT GTT AAA GTT AGT GAT C-3′) and Cest2 (5′-CAT AAA TCA ATG GAA ACA ACA ACA AG-3′) amplified a 395 bp stretch of the *nad*1 gene of *E. multilocularis*, primers Cest3 (5′-YGA YTC TTT TTA GGG GAA GGT GTG-3′) and Cest5 (5′-GCG GTG TGT ACM TGA GCT AAA C-3′) amplified a 267 bp stretch of the *rrnS* of *Taenia* spp., and primers Cest4 (5′-GTT TTT GTG TGT TAC ATT AAT AAG GGT G-3′) and Cest5 amplified a 117 bp stretch of the *rrnS* of *E. granulosus* (*s.l.*). Optimization of the original conventional PCR protocol [21] for use as a real-time PCR with DNA extracted from produce washes included systematic assessment of the following: primer concentration for Cest5 (range: 4 µM to 16 µM); optimal annealing temperature (range: 58 °C to 65 °C); annealing time (range: 30 s to 90 s); extension time (range: 10 s to 35 s); and addition of BSA (1×) or DMSO (1×) to the reaction mix. The final optimized reaction mix of 25 µl contained 1× Sso Fast™ EvaGreen Supermix (Bio-Rad, Hercules, CA, USA), 2 µM of primers Cest1, 2, 3 and 4, 4 µM of primer Cest5 (all primers: Integrated DNA Technologies, Coralville, IA, USA), 1× BSA (Sigma-Aldrich), and 2.5 µl of DNA. The optimized PCR cycling conditions were as follows: 3 min at 98 °C, followed by 40 cycles of denaturing at 98 °C for 15 s, annealing at 60  °C for 45 s, and extension at 72 °C for 10 s. Data collection was enabled at the annealing step. MCA began immediately following the last extension step and consisted of increasing the temperature from 65 °C to 95 °C by 0.2 °C increments with a 5 s hold at each step. All real-time PCR assays were performed with the CFX96™ Real-Time PCR Detection System and analyzed using the CFX Manager version 3.1. software (Bio-Rad Laboratories). All DNA samples were tested in duplicate. Positive (DNA of *E. multilocularis*, *E. granulosus* (*s.l.*) and *Taenia* spp.) and negative controls (negative extraction control and water) were included in all PCR runs. Standard curves for all PCR assays were prepared by ten-fold dilution of *T. pisiformis* DNA from 10^6^ eggs down to the equivalent of DNA from 10 eggs.

To determine the variation in melting temperatures for different taeniids, we repeated the PCR-MCA analysis and determined the average and standard deviation for each assessed parasite. The numbers of repeats included were: *n* = 28 for *E. granulosus* (G8/10); *n* = 30 for *E. multilocularis*; *n* = 110 for *T. pisiformis*; *n* = 41 for *T. saginata*; and *n* = 23 for *T. hydatigena*.

The analytical sensitivity of the real-time PCR protocol was assessed using DNA extracted with the FastDNA™ SPIN Kit for Soil from 10^6^ *T. pisiformis* eggs and then 10-fold dilutions down to the DNA equivalent of a single egg. Genomic DNA of *E. multilocularis*, *E. granulosus* (G8/10), *T. hydatigena* and *T. saginata* was adjusted to 10 ng/µl each and similarly tested in 10-fold dilutions to determine the analytical sensitivity of the PCR protocol for each species. Mixtures of target DNA adjusted to 10 ng/µl each were also used to test for preferential amplification in the PCR.

### Sequencing

Sanger sequencing of PCR products was performed by a commercial service provider (Plant Biotechnology Institute, National Research Council, Saskatoon, Canada). Amplified products were prepared for sequencing using the QIAquick PCR Purification Kit (Qiagen) following the manufacturer’s instructions. Primers Cest3 and 5 for *Taenia* spp., Cest1 and 2 for *E. multilocularis*, and Cest4 and 5 for *E. granulosus* (*s.l.*) were used in the sequencing reactions at a concentration of 5 µM each. Sequences were assembled and trimmed using Clone Manager Professional 9 (SciEd Software) and then compared to the NCBI nucleotide sequence database using NCBI BLAST (https://blast.ncbi.nlm.nih.gov/Blast.cgi?PAGE_TYPE=BlastSearch).

### Statistical analyses

We assessed the quantitative differences in performance between wash solutions and between DNA extraction kits using linear mixed models [22]. Wash solutions were compared after spiking five or 500 eggs of *T. pisiformis* on lettuce and after spiking 100 eggs of *T. pisiformis* on strawberries. A model was built for each spiking protocol using the following formula:

$$ Cq_{ijk} = \alpha + \beta \times wash_{i} + a_{j} + \varepsilon_{ijk} $$

where the quantification value (Cq) is a linear function of the intercept (*α*) plus the effect of the wash solution (β *× wash*) and the residuals *ε*. A random factor *a* was added to account for unexplained variation between sample duplicates. The index *i* refers to wash solutions, *j* to each sample and *k* to each sample duplicate. To account for heteroscedasticity, each level of wash solution was allowed to have a different residual variance, hence $var\left( {\varepsilon_{ijk} } \right) = \sigma_{i}^{2}$.

The sensitivity of DNA extraction kits and the difference between the kits was assessed using the following formula:

$$ Cq_{ijk} = \alpha + \beta_{1} \times \log \left( {egg} \right) + \beta_{2} \times kit_{i} + a_{j} + \varepsilon_{ijk} $$

where the Cq is now a function of the intercept (*α*) plus the log of the number of spiked eggs (β_1_ *× log(egg)*), the effect of the extraction kit (β_2_ *× kit*), the random factor *a*, and the residuals *ε*. The index *i* refers to the extraction kit, *j* to each sample and *k* to each sample replicates. To account for heteroscedasticity, the variance of the residuals was modelled as σ^2^ multiplied with the power of the absolute value of the fitted Cq value, hence $var\left( {\varepsilon_{ijk} } \right) = \sigma_{i}^{2} \times \left| {\widehat{{Cq_{ijk} }}} \right|^{2\delta }$.

Level of significance was set at *P* < 0.05. Model fit was assessed by inspection of the residuals, alone, against fitted values, and against all explanatory variables. Normal distribution of the residuals was checked using QQ-plots and Shapiro-Wilk test. Data exploration and analysis were performed with the R software, version 3.5.3 [23], with additional packages *ggplot2* [24] and *nmle* [25].

## Results

### Evaluation of wash solutions

Combined results for all spiking experiments are summarized in Table 1. For romaine lettuce samples spiked with 500 *T. pisiformis* eggs, washing with glycine solution resulted in slightly lower Cq values than 0.1% Alconox, but the difference was not significant (Table 2, *P* = 0.0501, Additional file 1: Table S1). Sodium pyrophosphate resulted in significantly higher Cq values than both glycine and 0.1% Alconox (Table 2, *P* < 0.001, Additional file 1: Table S2). Alconox solution significantly outperformed glycine solution at the lowest spiking level in romaine lettuce (five eggs) (Table 2, *P* < 0.001, Additional file 1: Table S2) and was therefore used in the final protocol. Strawberry samples spiked with 100 eggs were not reliably detected using glycine or sodium pyrophosphate, respectively, whereas all Alconox-washed samples were correctly identified as positive (Table 2). Therefore, comparison of Cq values between wash protocols was not undertaken and only 0.1% Alconox was used in further experiments with strawberries.

**Table 1:** Combined results of all spiking experiments with romaine lettuce and strawberries

Number of eggs spiked	Romaine lettuce		Strawberries	
	Positive/Total	Mean Cq^a^	Positive/Total	Mean Cq^b^
500	30/30	26.49	nd	na
100	16/16	29.09	14/18^c^	28.09
50	15/15	29.95	1/1	30.09
10	12/12	34.05	9/9	32.36
5	21/21	34.2	10/10	32.8
0	0/9	na	0/8	na

^a^Glycine and 0.1% Alconox only

^b^0.1% Alconox only

^c^3 of 6 samples washed with glycine and 1 of 6 samples washed with sodium pyrophosphate were falsely negative

*Notes*: All lower spikes for strawberries were washed with 0.1% Alconox

*Abbreviations*: Cq, quantification cycle; nd, not done; na, not applicable

**Table 2:** Critical experiments for the selection of the best wash solution to recover *T. pisiformis* eggs from romaine lettuce or strawberries

Produce	No. of eggs spiked	0.1% Alconox		Glycine buffer		Sodium pyrophosphate	
		Positive/Total	Mean Cq	Positive/Total	Mean Cq	Positive/Total	Mean Cq
Lettuce	500	10/10	26.72	10/10	26.26	10/10	27.73*
Lettuce	5	10/10	33.37	11/11	35.47**	nd	nd
Strawberries	100	6/6^a^	28.08	3/6	29.18	5/6	29.78

^a^No comparison of Cq values between wash solutions was undertaken for strawberries, since only 0.1% Alconox correctly identified all samples as positive

*Note*: All lower spikes for strawberries were washed with 0.1% Alconox

*Significantly higher than Cq values for 0.1% Alconox (*P* = 0.0001) and glycine buffer (*P* < 0.0001)

**Significantly higher than Cq values for Alconox (*P* < 0.0001)

### Comparison of DNA extraction kits

**Fig. 1:**
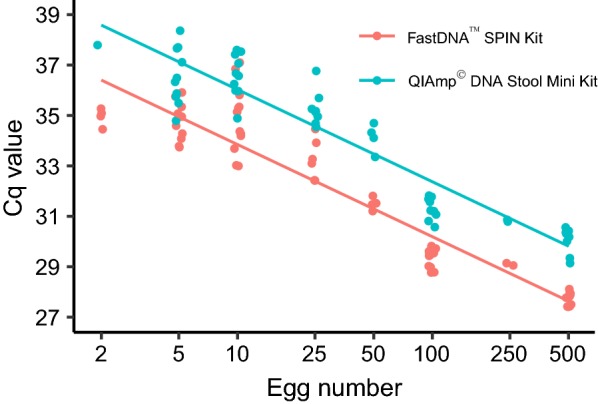
Comparison of two DNA extraction kits to extract DNA from *Taenia pisiformis* eggs suspended in negative produce wash. Quantification cycle (Cq) values for each PCR are depicted as individual dots and a regression line is shown for each extraction kit. Cq values for the FastDNA™ SPIN Kit for Soil were significantly lower than for the QIAamp® DNA Stool Mini Kit (*P* < 0.001)

The comparison of the two extraction kits applied to *T. pisiformis* eggs spiked into negative produce washes demonstrated that the FastDNA™ SPIN Kit for Soil method was superior to the modified QIAamp® DNA Stool Mini Kit method (Fig. 1). With both methods, the limit of detection was two eggs, with 1 of 5 and 2 of 5 samples positive using the QIAamp® DNA Stool Mini Kit and the FastDNA™ SPIN Kit for Soil, respectively (Additional file 1: Table S3). However, DNA samples extracted using the FastDNA™ SPIN Kit for Soil yielded significantly lower Cq values in real-time PCR (*P* < 0.001, Additional file 1: Table S4). Thus, the FastDNA™ SPIN Kit for Soil was used in the final protocol. Interestingly, the FastDNA™ SPIN Kit for Soil did not perform as well for taeniid eggs suspended in PBS for the extraction as for eggs suspended in produce wash (data not shown).

### Analytical sensitivity of the real-time PCR-MCA

**Fig. 2:**
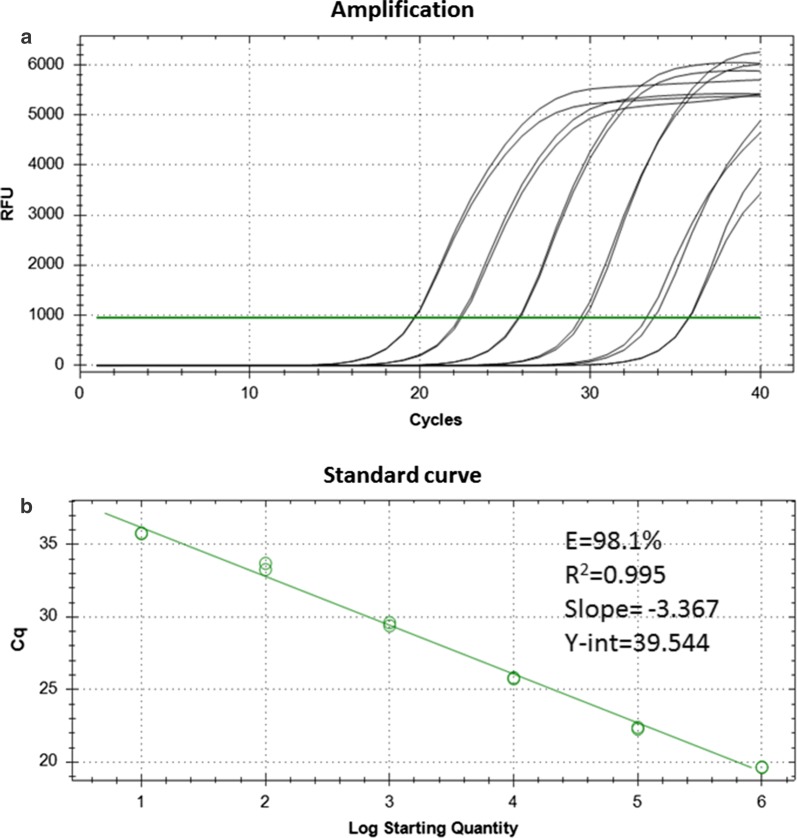
Amplification plot (**a**) and standard curve (**b**) generated from quantification cycle (Cq) values for serially diluted *T. pisiformis* DNA extracted from 10^6^ eggs

The analytical sensitivity of the real-time PCR-MCA as established using gDNA from different adult cestodes was 0.1 ng/reaction for *E. granulosus* (G8/10), 0.01 ng/reaction for both *T. saginata*, and *T. hydatigena*, and 1 pg/reaction for *E. multilocularis* (data not shown). Approximately 50% of the samples of DNA equivalent to one egg of *T. pisiformis* were detected by the PCR (data not shown). Therefore, one egg was regarded as the analytical limit of detection for *T. pisiformis*. The amplification plot and standard curve for DNA from 10^6^ to 10 *T. pisiformis* eggs are shown in Fig. 2. All produce samples spiked with five eggs were positive (Table 1). Lower spiking numbers were not attempted because of the difficulty in accurately pipetting the required number of eggs per sample. Reliable Sanger sequencing of the amplicons was achieved with the respective Cest primers, and BLAST analysis allowed species-identification and confirmation of specific amplification for all tested *E. granulosus* (G8/10), *E. multilocularis*, and *Taenia* spp., as well as for *Dicrocoelium* sp. (see ‘Analytical specificity of the real-time PCR-MCA’ below).

### Melting curve analysis

**Fig. 3:**
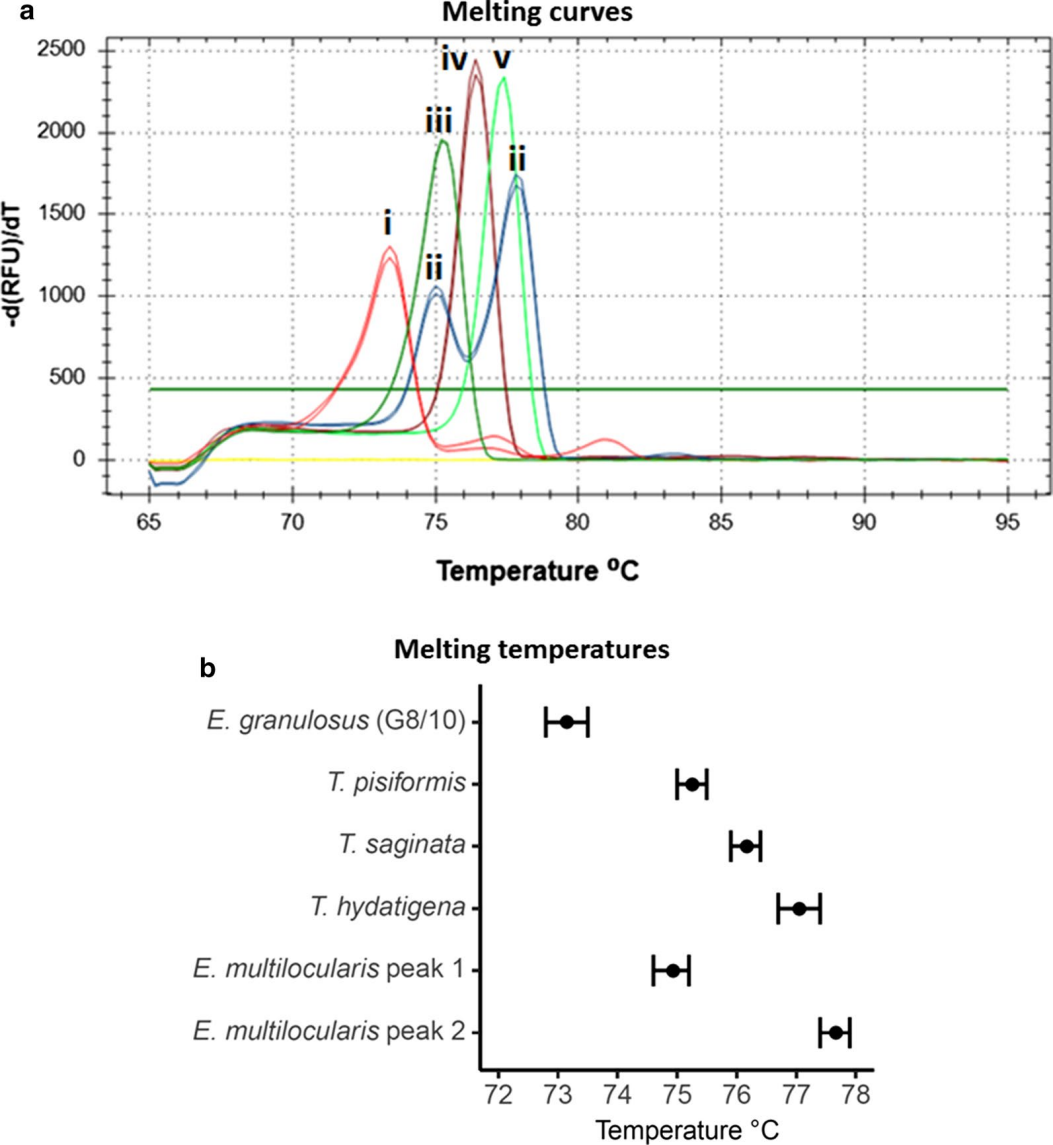
Melting curves of amplification products of different taeniids and average melting temperatures. **a** Melting curves for *Echinococcus granulosus* (G8/10) (red, i), *E. multilocularis* (blue, ii), *Taenia pisiformis* (dark green, iii), *T. saginata* (maroon, iv), and *T. hydatigena* (light green, v). Negative extraction control is the yellow line. **b** Average melting temperatures ± 2 SD are shown for each assessed species. Note that there is no overlap between *E. granulosus* (G8/10) or any of the *Taenia* spp. The two peaks of *E. multilocularis* overlap with *T. pisiformis* and *T. hydatigena*, respectively

*Echinococcus granulosus* (G8/10) had a characteristic melting peak at 73.2 °C (± 2 SD: 72.8–73.4 °C), *E. multilocularis* consistently displayed a melting curve with two peaks with the lower one at 74.9 °C (± 2 SD: 74.6–75.2 °C) and the higher one at 77.7 °C (± 2 SD: 77.4–77.9 °C) (Fig. 3). *Taenia* spp. displayed single melting peaks that varied according to species; *T. pisiformis* had a melting peak at 75.3 °C (± 2 SD: 75–75.5 °C), *T. saginata* at 76.2 °C (± 2 SD: 75.9–76.4 °C), and *T. hydatigena* at 77.1 °C (± 2 SD: 76.7–77.4 °C) (Fig. 3). The peak melting temperatures ± 2 SD did not overlap for *E. granulosus* (G8/10) or any of the *Taenia* species. However, the melting peaks of *E. multilocularis* overlapped with the ranges for *T. pisiformis* and *T. hydatigena*, respectively, but were still easily recognizable as they displayed two peaks (Fig. 3b).

### Mix of target DNAs

If DNA of more than one species were mixed, identification of mixes of *E. granulosus* (G8/10) and *E. multilocularis* was possible (Additional file 2: Figure S1). Also, mixes of *E. granulosus* (G8/10) and *T. hydatigena* or *T. pisiformis* DNA could be identified as such (Additional file 2: Figure S1). However, mixes of *E. multilocularis* and *Taenia* spp. DNA, as well as a mix of *E. granulosus* (G8/10), *E. multilocularis* and *T. hydatigena* DNA, resulted in atypical melting curves that did not enable discrimination of the species involved (Additional file 2: Figure S1).

### Analytical specificity of the real-time PCR-MCA

Produce washes of samples spiked with large numbers of *Eimeria* spp., *Giardia* sp. and *Cyclospora cayetanensis* did not result in any amplification in the real-time PCR; neither did gDNA extracted from *Toxoplasma gondii* (types I, II, III and atypical), *Sarcocystis* sp., *Uncinaria* sp., *Toxascaris* sp., *Capillaria* sp., *Trichuris* sp. or *Alaria* sp. (data not shown).

Amplification was observed with gDNA from *Dicrocoelium* sp. The 667-bp amplification product was sequenced and the closest BLAST hit with the highest query coverage of 98% had only 72% identity to a contig (GenBank: LK573795.1) obtained in a whole genome sequencing project for *D. dendriticum*. Other hits were represented by exclusively variable size fragments located in different contigs of this whole genome data set and also displayed comparatively low similarity levels. However, the melting curve profile of this product was distinct from those of the taeniids (Additional file 3: Figure S2).

**Fig. 4:**
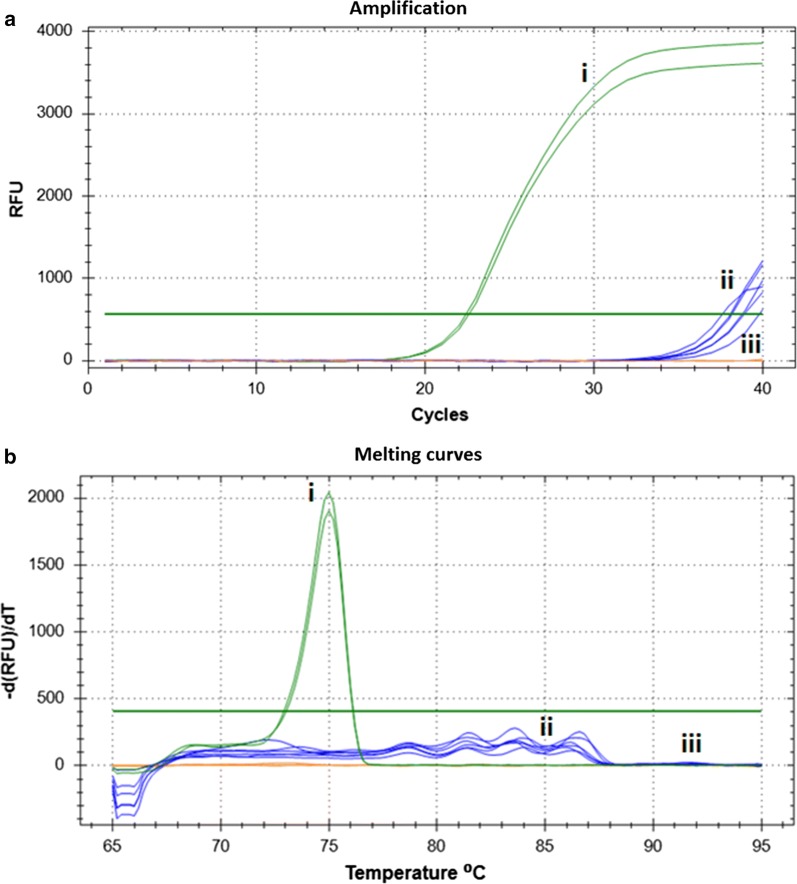
Spurious late amplification and melting profiles of negative produce washes. Amplification profiles (**a**) and melting curves (**b**) of *Taenia pisiformis* spiked control sample (*n* = 1) (green, i), non-spiked produce washes (*n* = 3) showing late amplification (blue, ii), and non-spiked produce washes (*n* = 3) not amplifying (orange, iii)

DNA extracted from negative produce washes resulted in about 50% of the samples showing late spurious amplification with high melting temperatures that were readily discernable from the target melting curve profiles (Fig. 4). Sequencing of the amplicons did not identify any similar sequences in GenBank. The spurious amplification did not occur when target taeniid DNA was present.

## Discussion

Despite being considered highly important food-borne parasites, only very few studies are available that show recovery of taeniid eggs from produce destined for consumption [6, 8, 9, 26]. To facilitate future surveillance aimed at elucidating this potential public health risk, we set out to develop and evaluate a sensitive and robust method to isolate, detect and differentiate such contamination in leafy greens and berries. The protocol established in our study proved to be extremely sensitive with correct identification of samples spiked with as few as five eggs per sample. Temesgen et al. [26] also recently reported a detection limit of five *E. multilocularis* eggs per 30 g sample of raspberries, while others have reported sensitivity of 100 eggs per sample [8], and protocols for the detection of *C. cayetanensis* contamination have detection limits of about ten oocysts per sample [13], or higher [14].

One of the most crucial steps in screening produce for parasite contamination is the wash and concentration protocol used to elute the parasite stages from the matrix. We tested three wash solutions that had previously proved successful in removing protozoan oocysts from produce or from fecal matrices. Glycine buffer was used in a standardized and validated method to detect *Cryptosporidium parvum* oocysts on lettuce and raspberries [12]; sodium pyrophosphate was used to efficiently isolate *C. cayetanensis* oocysts from fecal matter [11]; while 0.1% Alconox, a common laboratory detergent solution, is the wash buffer used by the US Food and Drug Administration (FDA) to remove *C. cayetanensis* oocysts from produce [13, 17] and had also been shown to enhance recovery of *C. parvum* from produce [10]. In our study, 0.1% Alconox was the most effective wash solution for removing taeniid eggs from both lettuce and strawberries. We concentrated the taeniid eggs by centrifugation only. This was deliberate in order to minimize loss of eggs that might occur in additional purification steps like for example sieving or flotation. It is also consistent with the FDA protocol for *C. cayetanensis* [13] and other studies for detection of taeniid eggs [26]. However, this approach poses a potential challenge to the DNA extraction step, as contaminants and inhibitors are not necessarily removed by centrifugation only.

Two commercially available DNA extraction kits designed to work with difficult matrices, and offering the advantages of reliability and standardization, were used to extract DNA from the robust thick-walled taeniid eggs in our study. We compared the two most successful commercial kits of a similar study targeting protozoan oocysts [27], namely the FastDNA™ SPIN Kit for Soil and the QIAamp® DNA Stool Mini Kit. In our study, the FastDNA™ SPIN Kit for Soil outperformed the QIAamp® DNA Stool Mini Kit for the detection of *Taenia* sp. DNA from eggs in produce wash. The FastDNA™ kit involved a bead-beating step, which has proven very efficient at disrupting the taeniid eggs, as shown by Maksimov et al. [28]. Interestingly, we observed that *Taenia* eggs suspended in PBS yielded lower levels of DNA than those suspended in negative produce wash. Therefore, we recommend using a negative produce wash as the carrier solution for eggs used as positive extraction controls in similar studies. It might be that certain steps in the protocol of this kit are not as efficient at releasing DNA in a “clean” solution such as PBS, relying on some amount of debris to provide a higher yield of DNA. While there are many advantages of commercial kits, one drawback is the potential discontinuation of a product; which happened with the QIAamp® DNA Stool Mini Kit in the course of this study.

The methods employed by Lass et al. [8, 9] specifically targeted *E. multilocularis*, whereas Federer et al. [6] used a conventional multiplex PCR [21], originally developed for the identification of taeniid eggs in fecal samples from carnivores, to simultaneously detect and discriminate between *E. granulosus* (*s.l.*), *E. multilocularis*, and *Taenia* spp. As all of these parasites may be relevant from a food safety perspective, we adapted the conventional multiplex PCR method targeting mitochondrial DNA used in [21], adapted it as a real-time PCR and optimized the reaction mix for use on DNA extracted from produce washes. The addition of MCA allowed for identification and differentiation of the *E. granulosus* species complex, *E. multilocularis*, as well as all tested *Taenia* spp. A multiplex real-time PCR with fluorescence MCA has been used for the detection and differentiation of coccidian parasites [16]. This latter method is currently being used as a screening tool for the surveillance of produce in Canada [14]. It also offers the advantage of being a closed-tube method, where no post-amplification handling of the reactions is necessary. This reduces the cross-contamination potential and is an advantage over nested PCR approaches. We had also previously assessed other multiplex PCRs, such as the method developed by Boubaker et al. [29], or the *12S* rRNA PCR by Roelfselma et al. [30], but those primers either lacked the necessary sensitivity or specificity to be used on produce washes (data not shown).

The primers used in our study cannot be considered as taeniid-specific, but should rather be regarded as cestodes-specific, or, considering the potential amplification of *Dicrocoelium* DNA, even flatworm-specific. In addition to the amplification of DNA of *Mesocestoides*, *Dipylidium* and *Diphyllobothrium* observed in the original publication of the multiplex PCR [21], we found amplification of *Dicrocoelium* sp. DNA. The amplified product produced a readily discernible melting temperature peak. Although the BLAST analysis of the sequence produced hits with exclusively variably sized fragments of different whole-genome sequencing contigs of *Dicrocoelium dendriticum*, the sequence homology levels were comparatively low. This and the availability of the complete mitochondrial genome sequences of *D. dendriticum* in GenBank suggest an off-target amplification of an area of the nuclear genome that is absent in the available whole genome data set, or correspond to a misassembled fragment. DNA samples from other flatworm species will need to be tested in future experiments to determine whether they are amplified and what their melting characteristics are. It should also be noted that *Alaria* sp. did not amplify in the present study.

Spurious late amplification of background DNA repeatedly occurred in negative produce washes. Initially, these random amplifications were present in virtually all negative reactions. The increase in annealing temperature, addition of BSA to the reaction mix and subtle changes in primer concentrations and the cycling protocol compared to the original PCR [21] resulted in enhanced analytical specificity of the present protocol, without reducing its analytical sensitivity. Melting temperatures, as well as the number and shape of the melting peaks, were very specific for all taeniids tested in the present study and considerably increased the specificity of the assay. Still, it is crucial that appropriate controls are included in all PCR runs in order to directly compare the melting profiles of unknown samples with the controls. Furthermore, the amplification products were easily sequenced using the original primers. This enabled species identification of *Taenia* spp.

Although the presence of more than one parasite species on a produce sample is not often observed by the protozoan contamination surveillance programme, it is possible. Therefore, we also tested mixed target DNAs and found that the presence of *E. granulosus* (*s.l.*) and *E. multilocularis* or *E. granulosus* (*s.l.*) and *Taenia* spp. can be detected by the real-time PCR-MCA. However, the method does not reliably identify the simultaneous presence of *E. multilocularis* and *Taenia* spp. DNA, or of all three target DNAs. Those reactions resulted in oddly-shaped melting curves within the temperature limits established for the target species. If contamination with *Echinococcus* spp. was the main target of investigation, the use of only the primers targeting *E. granulosus* (*s.l.*) and *E. multilocularis* might be an option to increase the resolution power of the assay. It is noteworthy that the primers targeting *Taenia* spp. also amplify DNA of other cestodes [21] and possibly of *Dicrocoelium* sp. (this study). Depending on the purpose of testing, the primers could be adapted accordingly.

The protocol to recover and identify taeniid eggs from lettuce and strawberries developed in our study proved to be sensitive and reasonably specific, especially when confirmatory sequencing was performed. However, more produce matrices need to be tested to evaluate whether the protocol works on different product types. Furthermore, in future studies, other parasite species with the potential to amplify in the PCR should be included to further characterize the performance parameters of this assay. If a future application of the method included targeting particular species and not all taeniids, the EvaGreen system could potentially be replaced by specific primer-probe pairs to enhance specificity. Regardless, any such protocol would need to be assessed using field samples to determine diagnostic sensitivity and specificity before it could be implemented for routine diagnostic or surveillance purposes.

## Conclusions

There is a need for sensitive and robust methods to test leafy greens and berries for contamination with taeniid eggs [5]. Based on the existing protocols available to detect protozoan contamination, we developed and standardized a protocol for the detection and differentiation of taeniid contamination in fresh produce. The novel protocol had a very high analytical sensitivity of five eggs spiked per sample. Although the real-time PCR used in this protocol is specific for cestodes, the MCA allowed distinguishing between *E. granulosus* (*s.l.*), *E. multilocularis* and *Taenia* spp. Species identification for *Taenia* spp. was possible through amplicon sequencing. This novel standardized protocol could prove to be an effective tool to assess the risk of exposure of consumers to taeniid eggs in fresh produce.

## Supplementary information

**Additional file 1: Table S1.** Effect of the wash solution on the Cq value after spiking 500 eggs of *T. pisiformis* on lettuce. *n* = 60, groups *n* = 30, AIC = 38.761. **Table S2.** Effect of the wash solution on the Cq value after spiking five eggs of *T. pisiformis* on lettuce. *n* = 42, groups *n* = 21, AIC = 95.577. **Table S3.** Comparison of two DNA extraction kits to extract DNA from *T. pisiformis* eggs suspended in negative produce wash. **Table S4.** Effect of the DNA extraction kits on the Cq values after suspension of *T. pisiformis* eggs in negative produce wash. *n* = 117, groups *n* = 32, AIC = 246.304.

**Additional file 2: Figure S1.** Melting curves for mixed DNA samples. DNA samples were diluted to 10 ng/µl each. **a** *E. granulosus* (G8/10) and *E. multilocularis*. **b** *E. granulosus* (G8/10) and *T. hydatigena*. **c** *E. granulosus* (G8/10) and *T. pisiformis.* **d** *E. multilocularis* and *T. hydatigena.* **e** *E. multilocularis* and *T. pisiformis.* **f** *E. granulosus* (G8/10), *E. multilocularis* and *T. hydatigena.* X-axis: temperature in Celsius.

**Additional file 3: Figure S2.** Amplification and melting curve of *Dicrocoelium dendriticum*. **a** Amplification plots for *T. saginata* (red, i), and *Dicrocoelium* sp. (green, ii). **b** Melting curves for *T. saginata* (red, i), and *Dicrocoelium* sp. (green, ii).

## Data Availability

All relevant data supporting the conclusions of this study are included in the article and its additional files. The raw data are available from the corresponding author upon reasonable request.

## Abbreviations

PCR-MCA: polymerase chain reaction with melt curve analysis
