# Supplementary material for: A novel protocol to isolate, detect and differentiate taeniid eggs in leafy greens and berries using real-time PCR with melting curve analysis
Source: Parasit Vectors. 2019 Dec 18;12:590. doi: 10.1186/s13071-019-3834-8 (PMC6918723; doi:10.1186/s13071-019-3834-8)
Supplement: Supplementary file 1 — Additional file 1: Table S1. Effect of the wash solution on the Cq value after spiking 500 eggs of T. pisiformis on lettuce. n = 60, groups n = 30, AIC = 38.761. Table S2. Effect of the wash solution on the Cq value after spiking five eggs of T. pisiformis on lettuce. n = 42, groups n = 21, AIC = 95.577. Table S3. Comparison of two DNA extraction kits to extract DNA from T. pisiformis eggs suspended in negative produce wash. Table S4. Effect of the DNA extraction kits on the Cq values after suspension of T. pisiformis eggs in negative produce wash. n = 117, groups n = 32, AIC = 246.304. [file 13071_2019_3834_MOESM1_ESM.docx]

**Additional file 1: Table S1.** Effect of the wash solution on the Cq value after spiking 500 eggs of *T. pisiformis* on lettuce. N = 60, Groups = 30, AIC = 38.761

| Variable |  | Estimate | *se* | *df* | *t* | *P* |
| --- | --- | --- | --- | --- | --- | --- |
| Intercept |  | 26.724 | 0.161 | 30 | 166.045 | <0.001 |
| Wash solution |  |  |  |  |  |  |
| Alconox |  | Baseline |  |  |  |  |
| Glycine |  | -0.465 | 0.227 | 27 | -2.051 | 0.050 |
| Sodium pyrophosphate |  | 1.005 | 0.228 | 27 | 4.407 | <0.001 |

**Additional file 1: Table S2.** Effect of the wash solution on the Cq value after spiking five eggs of *T. pisiformis* on lettuce. N = 42, Groups = 21, AIC = 95.577

| Variable |  | Estimate | *se* | *df* | *t* | *P* |
| --- | --- | --- | --- | --- | --- | --- |
| Intercept |  | 33.372 | 0.218 | 21 | 153.213 | <0.001 |
| Wash solution |  |  |  |  |  |  |
| Alconox |  | Baseline |  |  |  |  |
| Glycine |  | 2.102 | 0.335 | 19 | 6.283 | <0.001 |

**Additional file 1: Table S3.** Comparison of two DNA extraction kits to extract DNA from *T. pisiformis* eggs suspended in negative produce wash

|  | **QIAamp® DNA Stool Mini kit** | |  | **FastDNA**™ **SPIN Kit for Soil** | |
| --- | --- | --- | --- | --- | --- |
| **# of eggs** | positive / total | avg. Cq of positives |  | positive / total | avg. Cq of positives |
| **500** | 6/6 | 30.09 |  | 8/8 | 27.73 |
| **250** | 1/1 | 30.82 |  | 1/1 | 29.32 |
| **100** | 6/6 | 31.36 |  | 7/7 | 29.36 |
| **50** | 2/2 | 34.12 |  | 2/2 | 31.50 |
| **25** | 4/4 | 35.27 |  | 3/3 | 33.27 |
| **10** | 7/7 | 36.59 |  | 8/8 | 34.69 |
| **5** | 5/5 | 36.55 |  | 5/5 | 34.75 |
| **2** | 1/5 | 37.79 |  | 2/5 | 34.94 |
| **0** | 0/4 | na |  | 0/4 | na |

avg.: average. na: not applicable

**Additional file 1: Table S4.** Effect of the DNA extraction kits on the Cq values after suspension of *T. pisiformis* eggs in negative produce wash. N = 117 , Groups = 32, AIC = 246.304

| Variable |  | Estimate | *s.* | *df* | *t* | *P* |
| --- | --- | --- | --- | --- | --- | --- |
| Intercept |  | 37.440 | 0.376 | 84 | 99.579 | <0.001 |
| Log(egg) |  | -1.575 | 0.095 | 30 | -16.619 | <0.001 |
| DNAExtraction kit |  |  |  |  |  |  |
| FastDNA™ SPIN Kit for Soil |  | Baseline |  |  |  |  |
| QIAamp® DNA Stool Mini kit |  | 2.174 | 0.070 | 84 | 31.065 | <0.001 |
