# Supplementary figures and images for: A novel protocol to isolate, detect and differentiate taeniid eggs in leafy greens and berries using real-time PCR with melting curve analysis
Source: Parasit Vectors. 2019 Dec 18;12:590. doi: 10.1186/s13071-019-3834-8 (PMC6918723; doi:10.1186/s13071-019-3834-8)

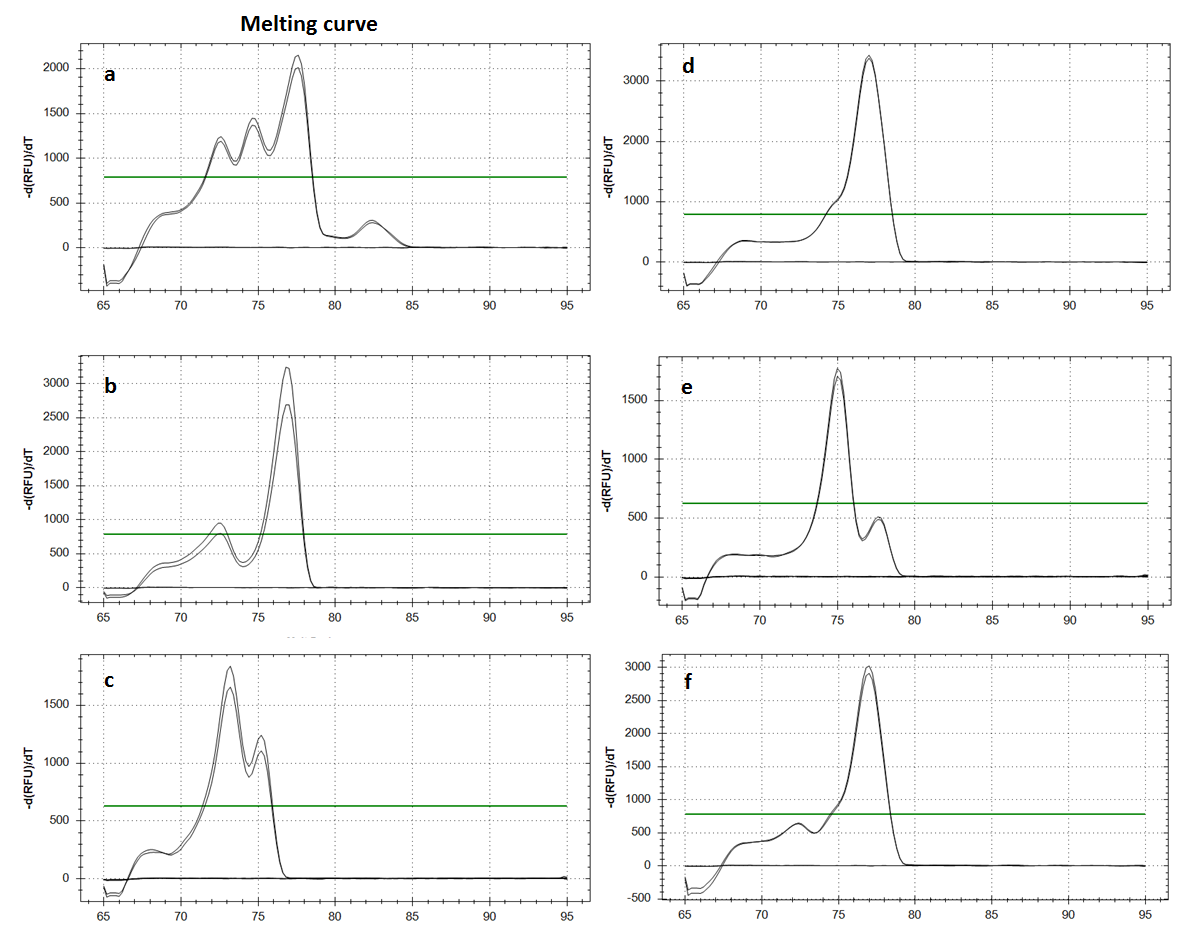

Supplement: Supplementary file 2 — Additional file 2: Figure S1. Melting curves for mixed DNA samples. DNA samples were diluted to 10 ng/µl each. a E. granulosus (G8/10) and E. multilocularis. b E. granulosus (G8/10) and T. hydatigena. c E. granulosus (G8/10) and T. pisiformis. d E. multilocularis and T. hydatigena. e E. multilocularis and T. pisiformis. f E. granulosus (G8/10), E. multilocularis and T. hydatigena. X-axis: temperature in Celsius. [file 13071_2019_3834_MOESM2_ESM.tif]

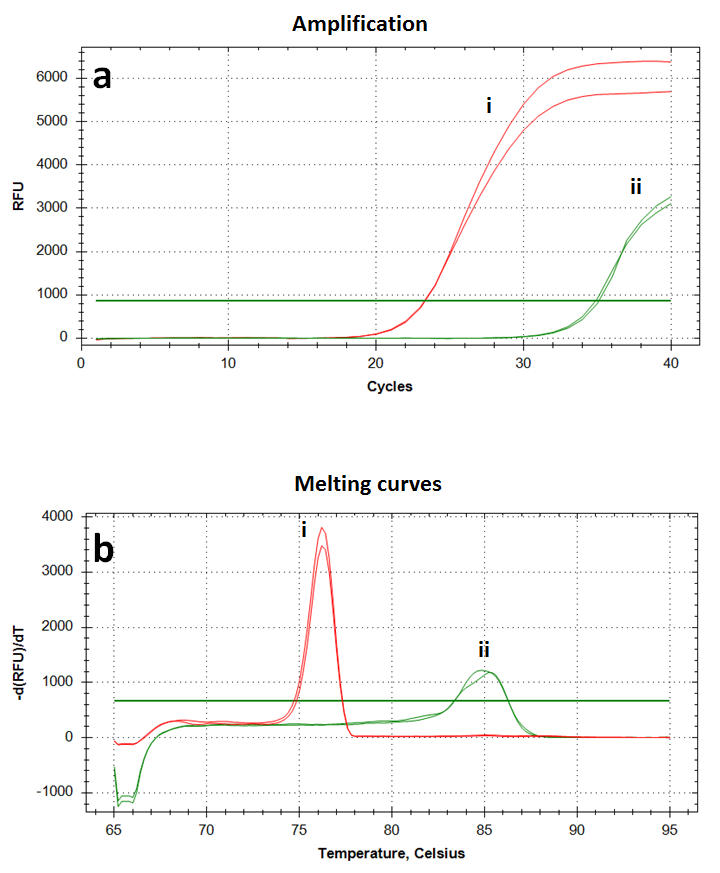

Supplement: Supplementary file 3 — Additional file 3: Figure S2. Amplification and melting curve of Dicrocoelium dendriticum. a Amplification plots for T. saginata (red, i), and Dicrocoelium sp. (green, ii). b Melting curves for T. saginata (red, i), and Dicrocoelium sp. (green, ii). [file 13071_2019_3834_MOESM3_ESM.tif]
